# Prevalence of CYP2D6 Genotypes and Predicted Phenotypes in a Cohort of Cambodians at High Risk for Infections with *Plasmodium vivax*

**DOI:** 10.4269/ajtmh.20-0061

**Published:** 2020-05-11

**Authors:** Michele D. Spring, Chanthap Lon, Somethy Sok, Darapiseth Sea, Mariusz Wojnarski, Soklyda Chann, Worachet Kuntawunginn, Thay Kheang Heng, Samon Nou, Montri Arsanok, Sabaithip Sriwichai, Pattaraporn Vanachayangkul, Jessica T. Lin, Jessica E. Manning, Krisada Jongsakul, Sathit Pichyangkul, Prom Satharath, Philip L. Smith, Lek Dysoley, David L. Saunders, Norman C. Waters

**Affiliations:** 1 US Army Armed Forces Research Institute of Medical Sciences, Bangkok, Thailand;; 2 The Henry M. Jackson Foundation for the Advancement of Military Medicine, Inc., Bethesda, Maryland;; 3 University of North Carolina-Chapel Hill, Chapel Hill, North Carolina;; 4 US National Institute of Allergy and Infectious Diseases, National Institutes of Health, Phnom Penh, Cambodia;; 5 Royal Cambodian Armed Forces, Phnom Penh, Cambodia;; 6 Walter Reed Army Institute of Research, Silver Spring, Maryland;; 7 National Malaria Program of Cambodia, Phnom Penh, Cambodia;; 8 U.S. Army Medical Research Institute of Infectious Diseases, Ft. Detrick, Maryland

## Abstract

Clinical failure of primaquine (PQ) has been demonstrated in people with CYP450 2D6 genetic polymorphisms that result in reduced or no enzyme activity. The distribution of CYP2D6 genotypes and predicted phenotypes in the Cambodian population is not well described. Surveys in other Asian countries have shown an approximate 50% prevalence of the reduced activity CYP2D6 allele *10, which could translate into increased risk of PQ radical cure failure and repeated relapses, making interruption of transmission and malaria elimination difficult to achieve. We determined CYP2D6 genotypes from 96 volunteers from Oddor Meanchey Province, Cambodia, an area endemic for *Plasmodium vivax*. We found a 54.2% frequency of the *10 allele, but in approximately half of our subjects, it was paired with a normal activity allele, either *1 or *2. The prevalence of *5, a null allele, was 9.4%. Overall predicted phenotype percentages were normal metabolizers, 46%; intermediate metabolizers, 52%; and poor metabolizers, 1%.

As control efforts reduce the malaria burden in Cambodia, the proportion of *Plasmodium vivax* to *Plasmodium falciparum* cases grows.^1^ This will necessitate widespread administration of radical cure treatment with primaquine (PQ) (or tafenoquine when available) to eliminate the dormant hypnozoites of *P. vivax* to prevent continued relapses and ongoing transmission within the community. Certain polymorphisms in the hepatic cytochrome P450 2D6 enzyme gene can lead to reduced or no metabolism of PQ into its active forms and can, therefore, negatively affect the efficacy.^2,3^ The CYP2D6 genotype, and the resulting predicted enzyme phenotype, varies by ethnicity,^4^ and understanding the population distribution can help assess the public health risk in areas where PQ is deployed.

There are more than 130 alleles of CYP2D6 currently identified (https://www.pharmvar.org/gene/CYP2D6, accessed October 11, 2019), and knowing an individual’s diplotype permits the prediction of one of four enzyme phenotypes: normal metabolizers (NMs), ultra-rapid metabolizers (UMs), intermediate (decreased/variable) metabolizers (IMs), and poor (null) metabolizers (PMs).^4^ A commonly used method for phenotype prediction uses the activity score-A (AS-A) developed by Gaedigk et al.,^5^ whereby each allele is assigned a score, ranging from 0 (no activity), 0.5 (reduced activity), to 1.0 (normal activity), and the two scores are added together. In March 2019, the Clinical Pharmacogenetics Implementation Consortium (CPIC), a U.S.-based advisory group for pharmacogenomic interpretations, assigned the *10 allele a score of 0.25 because “… it appears to be, in average, considerably lower compared to other decreased function alleles.”^6^ The CPIC also changed the interpretation of a diplotype AS-A of 1.0 from an NM to IM; thus, those from 0.25 to 1.0 fall in the IM category, 1.25–2.25 as NMs, > 2.25 as UMs, and those with a score of 0 as PMs.

Because there is limited information on CYP2D6 genotypes/predicted phenotypes from Cambodia, a country highly endemic for *P. vivax*, we retrospectively performed CYP2D6 genotyping on stored buffy coat samples from 96 volunteers who took part in two malaria studies in Oddor Meanchey Province, located in northern Cambodia, following re-consent for human DNA testing. Study A was a malaria cohort study with an all-species nested therapeutic efficacy study (TES) of dihydroartemisinin–piperaquine (DP) monotherapy, conducted with the Royal Cambodian Armed Forces from 2010 to 2011.^7^ *Plasmodium vivax* recurrences were treated with chloroquine, and PQ radical cure was given at study completion. Study B was a TES of DP for *P. falciparum*.^8^ CYP2D6 genotypes were determined from stored buffy coat samples using ×TAG^®^ CYP2D6 Kit v3 (Luminex^®^, Austin, TX) at the Laboratory for Pharmacogenomics and Personalized Medicine, Ramathibodi Hospital, Bangkok, Thailand. The CYP2D6 alleles detected by this assay include normal activity alleles (*1, *2, *35, and *39), null/no activity alleles (*3, *4, *5, *6, *7, *8, *11, and *15), and reduced activity alleles (*9, *10, *14b, *17, *29, and *41). Copy number variations of the 2D6 gene read as “DUP,” although the assay cannot distinguish which allele has more than one copy.

All 96 subjects from both Study A and B were successfully genotyped with allele frequencies shown in Table 1. The *10 allele was the most frequent (54.2%) followed by *1 (21.4%). The null allele *5 reached almost 10%, with only one person having the null *4 and none having *3, *6, and *7 or other rarer null alleles. Table 1 includes several other publications categorizing 2D6 allelic frequencies in neighboring countries: Thailand, Vietnam, the Karen ethnic minority group (from Myanmar) living in Thailand, and the two publications from Cambodia.^9–16^ In all, *10 was the most common allele and, throughout Asia, often reached 50% frequency.^17^ It was intriguing to see a higher percentage of the *5 null allele in our study population, although the sample size is small and from one province. As we continue to conduct malaria studies in Cambodia, we hope to better substantiate if the *5 allele may indeed be more frequent.

**Table 1:** CYP2D6 allelic frequencies in Cambodians and neighboring countries in the Greater Mekong subregion

CYP2D6 allele	Cambodia (*n* = 96)	Cambodia^9^ (*n* = 11)	Cambodia^10^ (*n* = 90)	Thai (Bangkok)^11^ (*n* = 721)	Karen (Tak)^12^ (*n* = 70)	Vietnam (Hanoi)^13,14^ (*n* = 86/*n* = 136)	Thai (Tak)^15,16^ (*n* = 82/*n* = 51)
*1	21.4	31.8	–	24.6	21.4	24/18.8	28.6/22.5
*2	10.9	4.5	–	10.8	32.9	11/7.4	17.1/29.4
*4	0.5	0	0	1.3	2.1	0.5/0.7	4.3/1
*5	9.4	4.5	–	6.7	2.9	5/8.1	/6.9
*9	0	0	–	0	–	0	–
*10	54.2	54.5	60.6	49.6	40	58.5/43.8	42.1/34.3
*14	0	–	–	0.1	–	/0.4	–
*17	0	0	7.7	0	–	/0	–
*29	0	0	–	0.1	–	/0	–
*35	0	–	–	0.1	–	/0	–
*36	–	–	–	0	0.7	/0.4	–
*39	0	4.5	–	0.2	–	/0	–
*41	3.6	0	–	6.5	–	1/0	7.9/5.9

Left column indicates the CYP2D6 allele. Allele color coding: bold values indicate no enzyme activity (AS-A allele haplotype score of 0); italic values, reduced activity (AS-A allele haplotype score of 0.25), underlined values, reduced activity (AS-A allele haplotype score of 0.5); and double underlined values, full enzyme activity (AS-A allele haplotype score of 1). At the time of our Cambodian study, allele 14 was split into 14a (null) and 14b (reduced). We tested for both. Reference 11 found 14b and reference 14 found 14a. Column titles are country (province) names. Values given in each box are percentages and adapted from references 9 to 16. Dashes indicate allele not tested or no report. Only reference 14 used sequencing for CY2D6 genotyping; data from other studies shown were those performed by PCR or Luminex^®^.

We also determined individual 2D6 diplotypes and predicted phenotypes using AS-A^5^ and found 52% classified as IMs, with 46% NMs, and 1% PMs (Figure 1A). One volunteer (1%) had a gene duplication which could lead to an interpretation of IM or NM. Overall, three volunteers were found to have gene duplications, but no one was predicted to have the UM phenotype. Within the 16 different genotypes (Figure 1A), the most common was *10/*10 in 29% of volunteers, followed by *1/*10 (25%) and *2/*10 (11%). Shown in Figure 1B–E are the 2D6 genotypes and predicted phenotypes adapted from the studies in Table 1 that listed diplotypes and used PCR or Luminex^®^ testing methods. Despite the high prevalence of the *10 allele, with an activity score of only 0.25, most studies had a majority of NMs because of frequent pairing with *1 or *2 normal allele. The very low rate of PMs is encouraging for malaria elimination, but the potential impact of high prevalence of IMs remains unclear. In the original case series at WRAIR, only one of three Caucasian IMs failed PQ treatment,^2^ but Baird et al. found a much higher risk of PQ failures in Indonesians designated as IMs.^18^ The IM-predicted phenotype encompasses a wide interval of activity scores (0.25–1), so an IM designation may not translate to equal risk for therapeutic failure. In a small pharmacokinetic (PK) study of PQ in healthy adults, PK parameters of IMs with AS-A of 0.25 (null allele/*10) were similar to PMs^19^; thus, for our study in Cambodia, 7% of our population could potentially be at risk for PQ failure due to no/severely reduced CYP2D6 activity.

**Figure 1.**
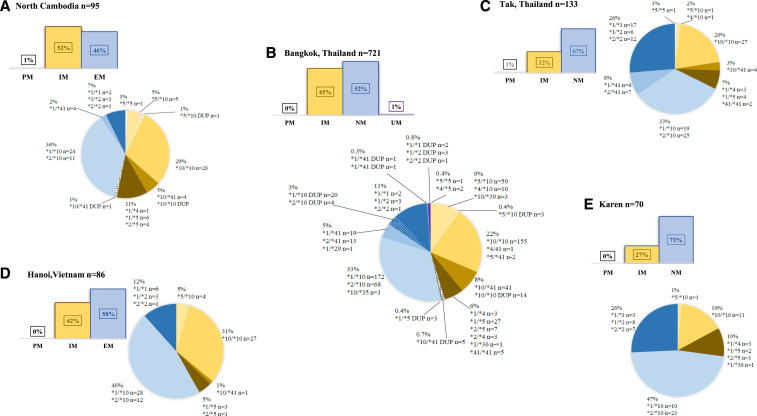
Percentages of CYP2D6 genotypes and predicted phenotypes. Pie charts for each location show percentages of each CYP2D6 diplotype activity score: 0, 0.25, 0.5, 0.75, 1, 0.25, 1.5, and 2. Poor metabolizer (PM, activity score-A [AS-A] of 0) is shaded in white with black border, intermediate metabolizers (IMs) are shades of yellow depending on AS-A (0.25, 0.5, 0.75, and 1), and normal metabolizers (NMs) are shaded in blues depending on AS-A (1.25, 1.5, and 2), and ultrarapid metabolizers (UMs) are shaded in purple. Volunteers with allele duplications that led to dual phenotype interpretations are in patterned slivers. Listed adjacent to each AS-A sliver are the corresponding diplotypes with the number of subjects typed. Bar graph insets show overall percentages of PMs, IMs, NMs, and UMs. (**A**) The current Cambodia study in Oddor Meanchey Province, (**B**) data adapted from reference 11 at Ramathibodi Hospital in Bangkok, (**C**) data adapted from references 15 and 16 with Thai subjects taking part in clinical trials in Tak Province in northwestern Thailand, (**D**) data adapted from reference 13 with subjects from Hanoi, Vietnam, and (**E**) data adapted from reference 12 with Karen subjects living at the Thai–Myanmar border area in Tak Province.

Of the 56 Study A volunteers who had re-consented to CYP2D6 genotyping, 44 had experienced a *P. vivax* infection over the approximate 4-month study period, with 16 of the 44 (36%) then having at least one vivax recurrence (range 1–4), as shown in Figure 2. The median time to recurrence after DP for primary *P. vivax* cases was 70 (mean 77, range 56–126) days and after CQ given for recurrence was 45 (mean 47, range 34–63) days. The pattern in Figure 2 reflects both the intense exposure of a military cohort working in a forested border area and the fast relapsing characteristics of vivax in Southeast Asia, reinforcing the necessity for a widespread, effective radical cure treatment to interrupt transmission. At the end of the DP efficacy study, PQ was administered as follows: 30 mg oral PQ daily for two weeks at weight-normalized doses at or above recommendations^20^ (6.36–9.03 mg/kg) in 35 subjects, and in the nine subjects who had glucose-6-phosphate dehydrogenase (G6PD) deficiency, 45 mg PQ once weekly for 8 weeks. During 6 months of monthly follow-up, five of the 44 volunteers (11.4%), all G6PD normal and with NM-predicted phenotype (AS-A 1.25, either *1/*10 (*n* = 4) or *2/*10 (*n* = 1)), presented with symptomatic patent *P. vivax* infection. The single PM subject in Study A did not relapse during the 6-month follow-up period. However, in the 4 months preceding PQ, the subject did not have any vivax recurrences; therefore, it is possible the subject did not harbor hypnozoites. All volunteers were part of a military population who remained deployed in the endemic area over the study period and follow-up; therefore, it is difficult to make definitive conclusions about PQ efficacy and CYP2D6 genotypes in this cohort.

**Figure 2.**
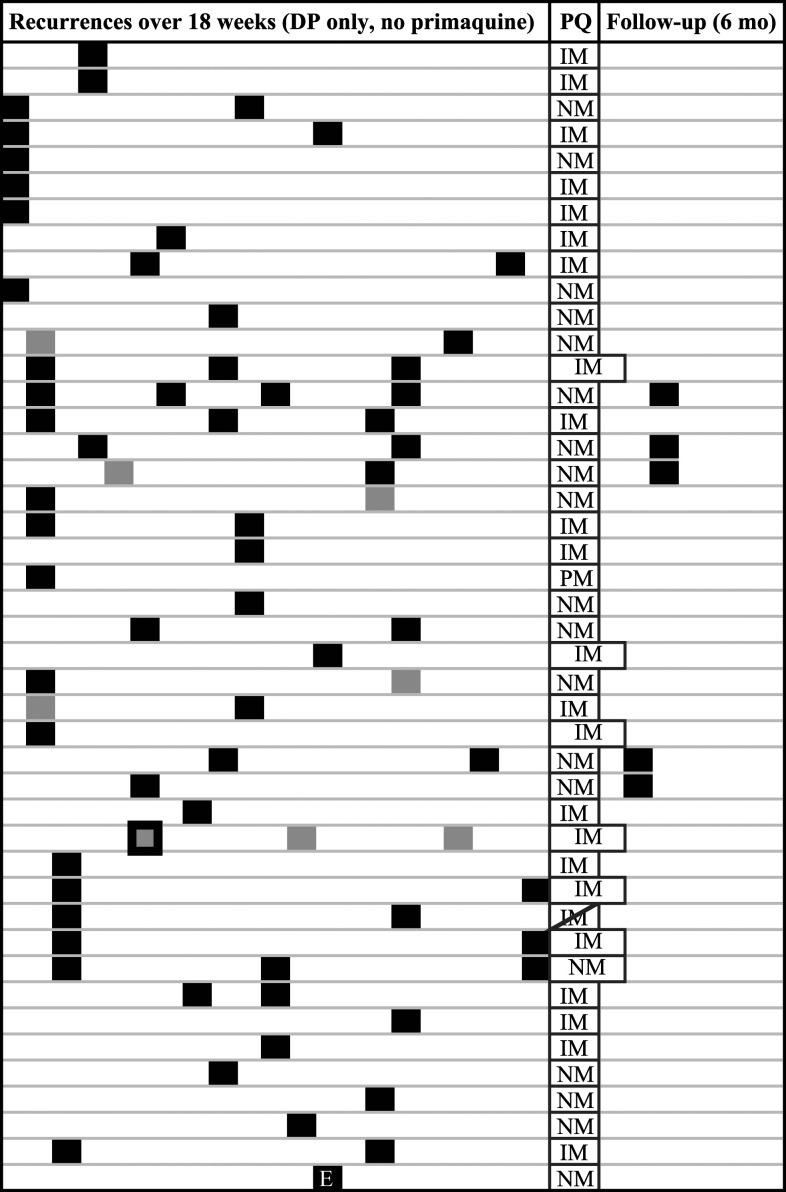
Pattern of vivax malaria recurrences in 44 Cambodian adults over an 18-week study period. Recurrences of malaria during the Study A active cohort are shown in which each primary case of malaria (all-species) was treated with dihydroartemisinin–piperaquine monotherapy and subsequent vivax recurrences with chloroquine. Each space represents 1 week. Black boxes represent *Plasmodium vivax* infections, gray boxes represent *Plasmodium falciparum* infections, and gray box with black border represents mixed *P. falciparum/vivax.* “E” stands for the one subject in this group who was enrolled in December, and thus had a shorter follow-up time. At conclusion of the active cohort, primaquine (PQ) radical cure was given. In PQ column, the predicted phenotype of that subject is listed: IM = intermediate metabolizer; NM = normal metabolizer; PM = poor metabolizer. The longer PQ boxes represent subjects who are glucose-6-phosphate dehydrogenase deficient and received 8 weeks of PQ. The diagonal line through one box represents a subject who did not complete the 2-week PQ course and dropped out. In the 6-month follow-up period, malaria recurrences are indicated, and each box represents 1 month.

In field studies aiming to establish a relationship between CYP2D6 and radical cure failure, PK parameters of PQ (not measured in Study A) may be helpful. The plasma area under the curve (AUC) of PQ may not consistently predict efficacy,^2,18^ but recently, 5,6-orthoquinone (5,6 OQ), the stable surrogate for unstable and presumed active metabolite, 5-hydroxyprimaquine, was measured in the urine of American adults of the NM predicted phenotype,^19^ whereas the IM and PM subjects had very low levels or none. Prospective longitudinal studies of *P. vivax* infections and relapses, inclusive of CYP2D6 genotype/phenotype and PQ PK to measure 5,6 OQ and other metabolites, may provide useful information on the pharmacogenomic liabilities. Recently, the Cambodian government announced plans to start widespread administration of the 2-week course of oral PQ for radical cure for elimination of dormant vivax hypnozoites. As the new policy is executed, this represents a unique opportunity to investigate PQ metabolism with respect to CYP2D6, particularly in populations at high risk for vivax infections and subsequent relapses.
